# Application of Biphase Complete Complementary Code for Ionospheric Sounding

**DOI:** 10.3390/s18092811

**Published:** 2018-08-26

**Authors:** Guobin Yang, Peng Duan, Chunhua Jiang, Tongxin Liu, Ting Lan, Zhengyu Zhao, Shuzhu Shi, Chen Xu

**Affiliations:** 1 School of Electronic Information, Wuhan University, Wuhan 430072, China; gbyang@whu.edu.cn (G.Y.); chuajiang@whu.edu.cn (C.J.); tongxin_liu@whu.edu.cn (T.L.); tinglan@whu.edu.cn (T.L.); zhaozy@whu.edu.cn (Z.Z.); xu-cm@whu.edu.cn (X.C.); 2 School of Remote Sensing and Information Engineering, Wuhan University, Wuhan 430079, China; shishuzhu@whu.edu.cn

**Keywords:** coherent interference, multi-station ionospheric sounding, complete complementary code

## Abstract

This paper illustrates the processes carried out for the application of biphase complete complementary code (CCC) for ionospheric sounding to address the coherent interference problem in multi-station ionospheric sounding. An algorithm to generate the biphase CCC is described, and the detailed process of waveform construction and signal processing is presented. Characteristics of the autocorrelation and cross-correlation are analyzed through simulations, and the technical feasibility of the application of CCC is explored. Experiments of ionospheric sounding with the CCC are also implemented to verify performance. Results demonstrate that the CCC performs well in multi-station ionospheric sounding, and is capable of eliminating the coherent interference in the network of ionosondes, compared to the conventional complementary code.

## 1. Introduction

Activities of ionospheric sounding started from 1926 when remote sensing of the ionosphere by means of radio sounding was described [1]. With the development of digital technology, analog ionosondes evolved into digital instruments and custom computer circuits capable of digital integration and digital spectrum analysis. Reinisch et al. [2] developed the Digisonde Portable Sounder (DPS) with a digital signal processor in 1997. In order to establish a denser network of ionosondes, the Low-Cost Ionosonde was proposed by Stamper et al. [3]. The latest digital ionosonde Digisonde-4D [4] implements the advanced capabilities of new digital radio frequency (RF) circuitry and embedded computers while preserving the basic principles of Digisonde family. A network of ionosondes equipped with Digidonde-4D has been established in Europe to monitor travelling ionospheric disturbances (TIDs) by simultaneous vertical and oblique incidence sounding [5]. Nowadays, the universal software radio peripheral (USRP) is implemented in ionospheric sounding owing to its flexibility in data processing, less rigidity of hardware structures, and generally larger data transmission channel. Chirp sounding with software-defined radio (SDR) technology was proposed in 2003 [6]. Barona et al. [7] illustrated the construction of an ionosonde from a USRP with GUN-Radio and MATLAB. In addition, BPSK signal shaping and processing with a USRP was proposed by Ivanov et al. [8], which implies that SDR technology has received increased attention.

In 1985, Wuhan University (Wuhan, China) collaborated with the University of Paris-Sud (Paris, France) in the field of ionosphere research. Since then, Wuhan University has been devoted to ionospheric study and the development of ionospheric sounding systems. In 2003, the Wuhan Ionospheric Oblique Backscattering Sounding System (WIOBSS) was successfully developed by the Ionosphere Laboratory at Wuhan University. The system is able to operate at three different modes, including the vertical, oblique, and oblique backscatter sounding functions. In order to achieve frequency and clock synchronization, the Global Positioning System (GPS) was applied to the receiver. Recently, the frequency band of WIOBSS has been extended from high frequencies to very high frequencies to improve system performance [9]. Besides, Shi et al. [10] proposed an oblique-incidence ionosonde and applied it to the localization of ionospheric irregularities. Among the WIOBSS family, intrapulse phase-coded pulses are used as the emission signal so that the receiver can receive the echoes during the intrapulse periods. In order to achieve a high signal processing gain, long pseudorandom sequences such as the m-sequences and the almost perfect autocorrelation sequences are adopted [11]. Yao et al. [12] reported ionograms obtained by WIOBSS with different waveforms, and determined that the interphase-coded complementary code has the best performance. However, when two stations perform ionospheric sounding synchronously with the same complementary code, both vertical and oblique ionograms can be observed, thereby raising the difficulty of automatically extracting ionospheric parameters from the present ionograms using the current methods [13]. On the other hand, coherent interference can exist if the complementary code is non-orthogonal. Furthermore, if there are more than two stations operating, the coherent interference problem becomes worse. Research on coherent interference suppression has a long history; the methods are generally based on spatial averaging [14] for narrow band signals or spectral averaging [15] for broadband signals. Adaptive beamforming methods have been proposed to overcome the problem [16,17,18]. However, these methods aimed at the cancellation of the interference, are not appropriate for application in ionospheric sounding.

The complete complementary code (CCC) was first proposed by Suehiro et al. [19] to study the N-shift cross-orthogonal sequences. Additionally, Suehiro designed a multicarrier CDMA architecture based on the orthogonal complementary code [20], which achieves high spreading efficiency and offers multiple access interference (MAI) free operation. Recently, the CCC has also been applied in the MIMO system because of its ideal correlation property [21,22,23]. Quadri-phase complete complementary pairs were adopted in the topside ionosphere sounder system [24]. In this study, a biphase CCC is developed for application to ionospheric sounding in order to overcome the aforementioned weaknesses associated with the ionospheric sounding network. We firstly present a brief definition of CCC and its generation algorithm, and then describe the construction of the waveform and the details of signal processing. Characteristics of the autocorrelation and cross-correlation are subsequently analyzed through simulations, followed by discussions of the experimental results of ionospheric sounding with the application of the complete complementary code.

## 2. Complete Complementary Code

For *N* *M*-order sequence sets {($S_{11}$, $S_{12}$,…, $S_{1M}$), ($S_{21}$, $S_{22}$,…, $S_{2M}$),…,($S_{N1}$,$\;S_{N2}$,…, $S_{NM}$)}, each element $S_{ij}$ is a sequence of length *L*, and ($S_{11}$, $S_{12}$,…, $S_{1M}$) is a subset. We can use a {*M*, *N*, *L*} sequence family to represent the sequence sets, where *M* is the number of sequences in a sequence set, *N* is the number of sequence sets and *L* is the length of the sequence $S_{n,m}$. When the sum of autocorrelation functions of each sequence in the same subset is zero except for zero shift, and the sum of crosscorrelation of two sequences of the same index in each two subsets is zero at all shifts, the {*M*, *N*, *L*} sequence family is considered to be complete complementary code. In other words, the {*M*, *N*, *L*} sequence meets the following Equations (1) and (2).

(1) $$\sum_{j=1}^{M}R_{S_{ij}}\left(\tau \right)=ML\delta \left(\tau \right),$$

(2) $$\sum_{j=1}^{M}R_{S_{ij},S_{kj}}\left(\tau \right)=0\left(i\neq k\right),$$

where $R_{S_{ij}}\left(\tau \right)$ is the autocorrelation function of the sequence $S_{ij},$ $R_{S_{ij},S_{kj}}\left(\tau \right)$ is the crosscorrelation function of the sequence $S_{ij}$ and $S_{kj}$, δ(τ) is a typical impulse function, and τ is the offset.

## 3. Waveform and Signal Processing

There are many kinds of generation algorithms for the complete complementary code [25,26,27,28]. Suchiro et al. [19] extended the idea of complementary sets to what is known as the complementary code, and proposed a method to construct complete complementary sequences of length $N^{2}$. Han [28] expanded the method, and proposed a further method to generate a set of length *MN* $\left(M,N\in Z^{+};N\leq M\right)$, and then an improved method to generate a set of length MN/P$\left(M,N,P\in Z^{+};N,P\leq M\right)$ [27], which offers a practical way to shorten the length of sequences in the subsets.

In this study, the CCC is generated through the method proposed by Han and Suchiro [28], which uses the unitary matrix to derive the matrix composed of CCC. In order to be applied to the biphase modulation system, the Hadamard matrix [29] is used as an alternative of the unitary matrix.

**Figure 1:**
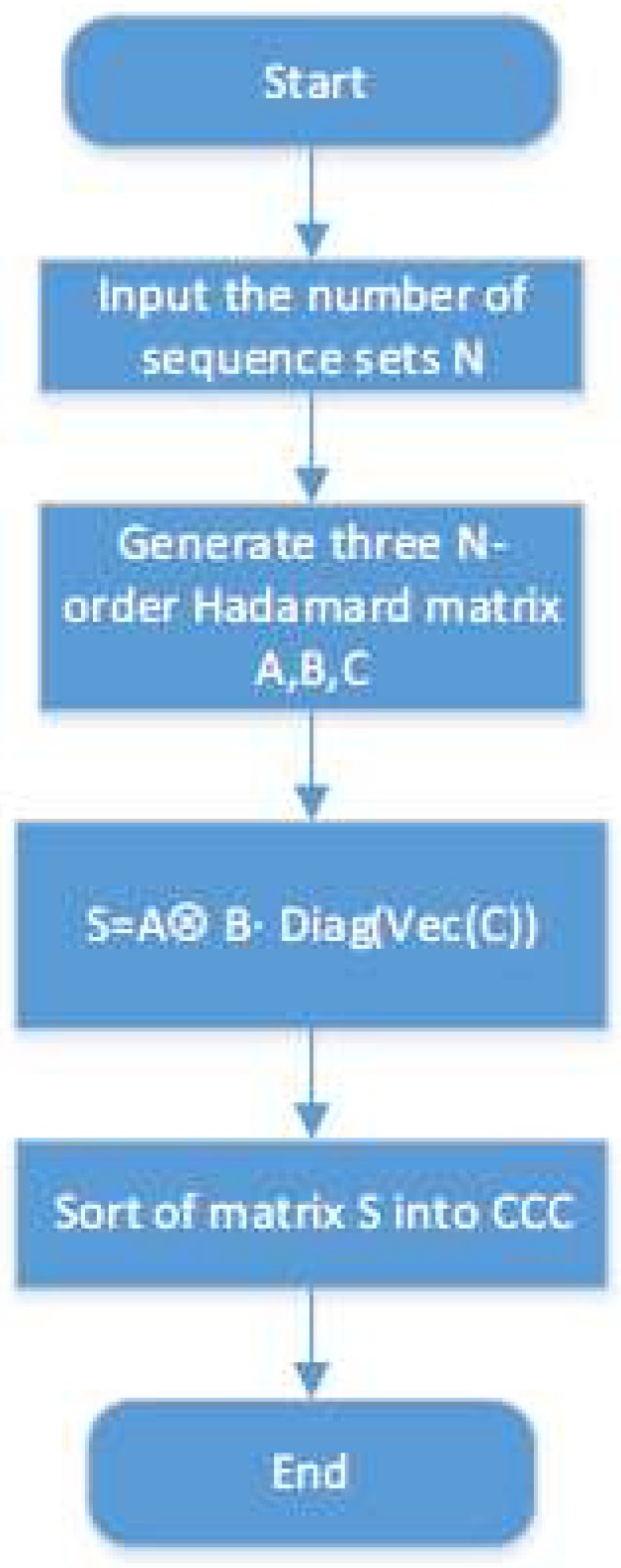
Generation method of {$N,N,N^{2}$} CCC.

Figure 1 shows the flow chart of {$N,N,N^{2}$} CCC’s generation. Firstly, the number of sequence sets *N* should be determined (*N* = $2^{k},k\in Z^{+}$). Secondly, three *N*-order Hadamard matrices should be generated; they can be indexed by A, B, C. Then, a $N^{2}\times N^{2}$ matrix *S* can be generated by a recapitulate formula. Three different matrix operations, Kronecker product, vectorization, and the “Diag” operator, are used in the formula. At last, we should transform the matrix *S* into sequence sets. Each row of *S* is a sequence, and each row of *N* is a subset. Table 1 shows a set of {4, 4, 16} complete complementary sequences. In Table 1, + and −, respectively representing +1 and −1, the four subsets are indexed by letters A, B, C, D, and the sequences in a subset are indexed by numbers 0, 1, 2, 3.

**Figure 2:**
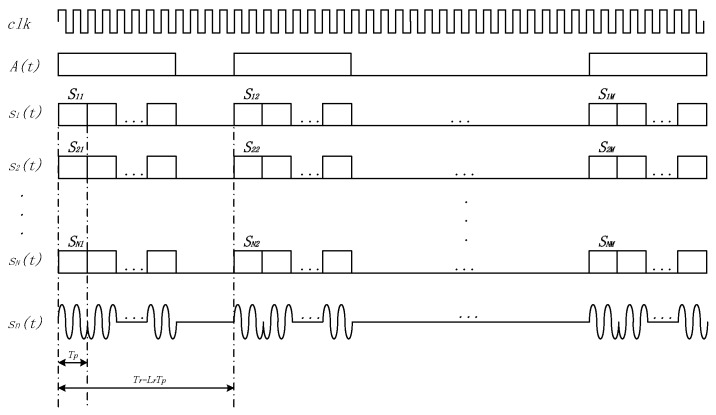
Waveform of a Single Cycle.

An intra-pulse biphase coding scheme was used in the WISS, where +1 and −1 represent the phase 0 and π respectively. According to the periodic correlation characteristics of the complete complementary code, sequences should be modulated in a certain order. Figure 2 shows the single-cycle transmitting waveform, where *A*(*t*) is the control sequence used to open or close the transmit channel; $s_{n}$(*t*) is a phase modulation signal based on the binary complete complementary code, $T_{p}$ represents the pulse width, and $T_{r}$ is the pulse repetition interval. 

The waveform of a single cycle is represented by Equation (3)

(3) $$s_{n}\left(t\right)=\frac{1}{\sqrt{ML}}\sum_{m=0}^{M-1}\sum_{i=0}^{L-1}S_{n,m}^{i}u\left(t-iT_{p}-mT_{r}\right)e^{j2\pi ft},$$

where $S_{n,m}^{i}$ is the *i*-th symbol of the sequence $S_{n,m}$, f is the carrier frequency, and u(t) is a rectangle pulse represented by Equation (4),

(4) $$u(t)\;=\{\begin{array}{c} \frac{1}{\sqrt{T_{p}}},\;0\leq t<T_{p}\\ 0,\;else \end{array}.$$

Suppose that there are two sites respectively transmitting $s_{1}\left(t\right)$ and $s_{2}\left(t\right)$, which means that the signal will travel in different propagation paths. Therefore, the delay time will be different, and they can be represented by $\Delta \tau_{1}$,$\;\Delta \tau_{2}$. Correspondingly, the Doppler shifts of the echoes are$\;\Delta f_{1}$, $\Delta f_{2}$. The echo signal can be demonstrated by Equation (5).

(5) $$\;s_{echo\;}\left(t\right)=\frac{A_{1}}{\sqrt{ML}}\sum_{m=0}^{M-1}\sum_{i=0}^{L-1}S_{1,m}^{i}u\left(t-iT_{p}-mT_{r}-\Delta \tau_{1}\right)e^{j2\pi \left(f+\Delta f_{1}\right)t}\;+\frac{A_{2}}{\sqrt{ML}}\sum_{m=0}^{M-1}\sum_{i=0}^{L-1}S_{2,m}^{i}u\left(t-iT_{p}-mT_{r}-\Delta \tau_{2}\right)e^{j2\pi \left(f+\Delta f_{2}\right)t},$$

where $A_{1}$, $A_{2}$ denote the amplitude of different waveforms.

By the matched filter of $s_{1}\left(t\right)$, the final output *s*(*t*) can be represented by Equation (6),

(6) $$\begin{array}{l} s(t)\;=\int_{t}^{t+T_{r}}s_{echo}\left(\tau \right)\cdot s_{1}^{*}\left(\tau -t\right)d\tau \\ \;=\frac{A_{1}}{\sqrt{ML}}\int_{t}^{t+T_{r}}\sum_{m=0}^{M-1}\sum_{i=0}^{L-1}S_{1,m}^{i}u\left(\tau -iT_{p}-mT_{r}-\Delta \tau_{1}\right)e^{j2\pi \left(f+\Delta f_{1}\right)\tau }\cdot s_{1}^{*}\left(\tau -t\right)d\tau \\ \;+\frac{A_{2}}{\sqrt{ML}}\int_{t}^{t+T_{r}}\sum_{m=0}^{M-1}\sum_{i=0}^{L-1}S_{2,m}^{i}u\left(\tau -iT_{p}-mT_{r}-\Delta \tau_{2}\right)e^{j2\pi \left(f+\Delta f_{2}\right)\tau }\cdot s_{1}^{*}\left(\tau -t\right)d\tau \\ \;=A_{1}\delta \left(t-\Delta \tau_{1}\right)e^{-j2\pi \Delta f_{1}t}\\ \;+\frac{A_{2}}{ML}\left[\int_{t}^{t+T_{r}}\sum_{m=0}^{M-1}\sum_{i=0}^{L-1}S_{2,m}^{i}u\left(\tau -iT_{p}-mT_{r}-\Delta \tau_{2}\right)\cdot S_{1,m}^{i}u\left(\tau -t-iT_{p}-mT_{r}\right)\cdot e^{-j2\pi \Delta f_{2}\tau }d\tau \right] \end{array}.$$

Due to the ideal auto-correlation property of CCC, the first part of Equation (6) is an impulse function, and the second part keeps zero in the entire time domain because of the orthogonality. Thus, we can extract the delay time $\Delta \tau_{1}$ and Doppler shift $\Delta f_{1}$. Additionally, if the echo signal is compressed by the matched filter of $s_{2}\left(t\right)$, $\Delta \tau_{2}$ and $\Delta f_{2}$ can be derived.

## 4. Feasibility Analysis

**Figure 3:**
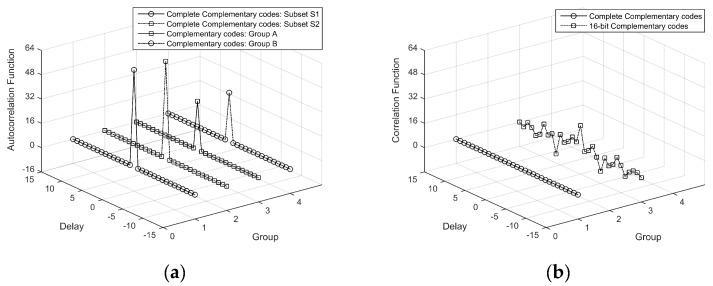
Analysis of {4, 4, 16} CCC and 16-bit complementary code: (**a**) Autocorrelation function (**b**) Cross correlation function.

In this section, characteristics of auto- and cross-correlation of the CCC and complementary codes are studied. Figure 3a shows the autocorrelation functions of two sets of non-orthogonal 16-bit complementary code and two subsets of a {4, 4, 16} complete complementary code. It can be noted from Figure 3a that the autocorrelation coefficients are zero except zero offset for the complementary code and CCC. Figure 3a indicates that both the complementary code and CCC have the ideal autocorrelation property. Since the coherent accumulation time of the {4, 4, 16} complete complementary code is twice of the complementary code, the autocorrelation gain is doubled in Figure 3a. Figure 3b shows the cross-correlation functions of the complementary code and CCC. The solid and dashed lines, respectively, represent the cross-correlation function between the two subsets of complete complementary code and the two non-orthogonal complementary codes. It can be seen from Figure 3b that the cross-correlation function of the CCC is better than that of the complementary code. Therefore, the ideal cross-correlation property of the complete complementary code makes it feasible for multiple stations to operate together without coherent interference.

For pulse compression radars, an ambiguity function χ(Δτ,Δf) is a two-dimensional function to measure the distortion of an echo signal, where Δτ is the group delay of transmitted signal and Δf is the Doppler shift. The narrowband ambiguity function is represented by Equation (7) in ionospheric sounding,

(7) $$\chi \left(\Delta \tau ,\Delta f,\right)=\int_{-\infty }^{+\infty }s\left(t\right)s^{*}\left(t-\Delta \tau \right)e^{j2\pi \Delta ft}dt,$$

where s(t) is the transmitted pulse waveform, and $s^{*}\left(t-\tau \right)e^{j2\pi ft}$ is the detected signal.

The ambiguity function of the complete complementary code is the sum of ambiguity functions of each sequence in a subset [30], which can be represented by Equations (8) and (9),

(8) $$\;\chi \left(\Delta \tau ,\Delta f\;\right)=\overset{M}{\underset{m=1}{\sum }}\int_{-\infty }^{+\infty }s_{m}\left(t\right)s_{m}^{*}\left(t-\Delta \tau \right)e^{j2\pi \Delta ft}dt\;=\frac{1}{ML}\sum_{m=1}^{M}\sum_{S=-\left(L-1\right)}^{L-1}\left|\chi_{1}\left(\Delta \tau_{1},\Delta f\right)\cdot \sum_{i=1}^{L-\left|S\right|}S_{n,m}^{i}S_{n,m}^{i+\left|S\right|}e^{j2\pi \Delta f\left(i-1\right)T_{p}}\right|,$$

where

(9) $$\chi_{1}\left(\Delta \tau_{1},\Delta f,\right)=\{\begin{array}{c} \frac{\sin\left[\pi \Delta f\left(T_{p}-\left|\Delta \tau_{1}\right|\right)\right]}{\pi \Delta fT_{p}},\;\left|\Delta \tau_{1}\right|\leq T_{p}\\ 0,\;else \end{array},$$

and $\Delta \tau_{1}=\left|\Delta \tau -ST_{p}\right|$.

**Figure 4:**
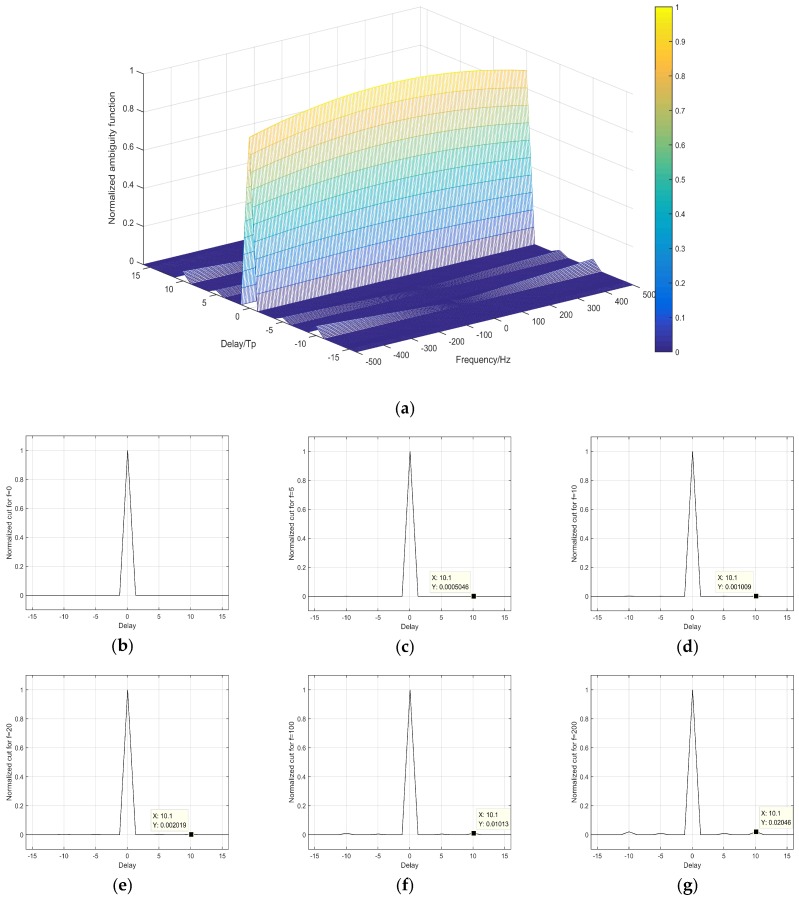
Normalized Ambiguity Function Analysis of Complete Complementary Code: (**a**) Normalized ambiguity function; Normalized ambiguity cut for (**b**) f = 0, (**c**) f = 5, (**d**) f = 10, (**e**) f = 20, (**f**) f = 100, (**g**) f = 200.

Figure 4a shows the normalized ambiguity function of a {4, 4, 16} complete complementary code. In consideration of the pulse width of waveform in WISS, $T_{p}$ is set 25.6 μs. Figure 4b–g is different cross sections of the ambiguity function, where f is the Doppler shift. When f = 0, the complete complementary code keeps an ideal autocorrelation property. When f = 5, the sidelobe peak of the normalized ambiguity function is 0.00025, about −36 dB, indicating that the complete complement code still has excellent autocorrelation characteristics. When f = 200, the sidelobe peak rises to 0.01, approximately −20 dB. It is seen that when Doppler shift fluctuates at a large range, CCC maintains low sidelobe peak level. Table 2 shows the sidelobe peak of ambiguity function with different Doppler shifts. As the Doppler shift grows, the sidelobe peaks of the m-sequence and Barker code hardly change, while those of the complementary code and CCC decrease considerably. Since the Doppler shift is generally less than 5 Hz in ionospheric sounding [31], it infers that the Doppler shift has little effect on the application of the complete complementary code.

Additionally, the number of subsets *M* and the length of sequence *L* cannot be too large. The longer the meta-code sequence, the larger the detection dead zone. Actually, the detection of the ionosphere is based on the smoothness of the ionosphere, and the state of the ionosphere is considered to be undisturbed within certain minutes. If the single cycle lasts too long, the detection validity can decrease significantly.

## 5. Experiments and Discussions

In this study, the WISS, installed at Ningqiang (105.20° E, 32.27° N) and Wuhan (114.22° E, 30.33° N), are utilized to carry out multi-station ionospheric sounding experiments to investigate the performance of the CCC application. The system operated at the HF-band to accomplish vertical and oblique incidence sounding. The ionosondes operated in the swept-frequency mode. Three-wire dipole and log-periodic antennas were used to transmit and receive the signals, respectively. In the present experiment, the biphase intrapulse waveform was coded by a 16-bit complete complementary code or a {4, 4, 16} complete complementary code. All the stations synchronously transmitted and received the signals through the global positioning system (GPS). Table 3 shows the parameters of the emission waveform.

**Figure 5:**
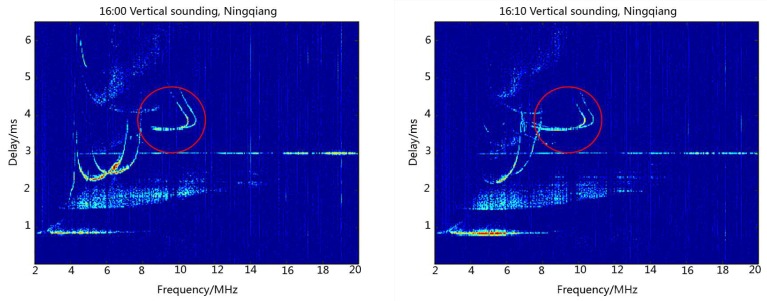

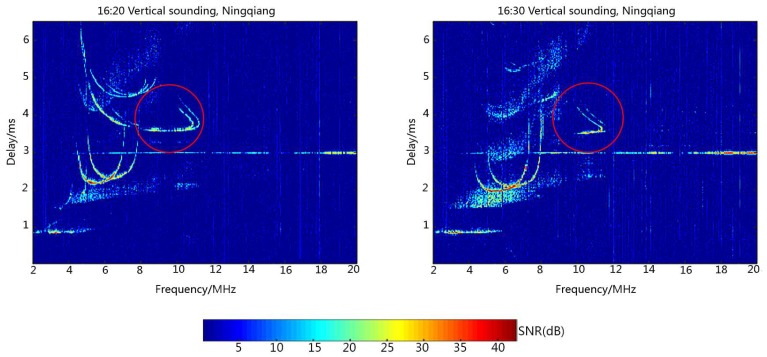
Multi-station sounding experiment using the same complementary code.

Figure 5 illustrates the ionograms recorded at Ningqiang on 23 June 2018 when the ionosondes installed at Ningqiang and Wuhan synchronously transmitted the same 16-bit complementary code at 14:00 Beijing time (BJT). Parameters of the ionosphere at Ningqiang can be obtained from ionograms. For example, based on the delay time, we can derive the virtual height of the E and F2 layers (h’E = 140 km, h’F2 = 330 km). The red circles in Figure 5 show the reflection signal echoes of the F-layer at the middle of the propagation path from Wuhan to Ningqiang. It is seen from Figure 5 that the echoes of the vertical sounding at Ningqiang and oblique sounding from Wuhan overlapped on the ionograms. Obviously, these kinds of ionograms are difficult to be scaled automatically using the current software.

**Figure 6:**
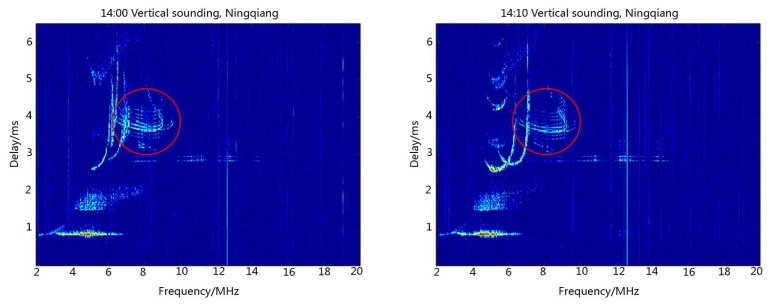

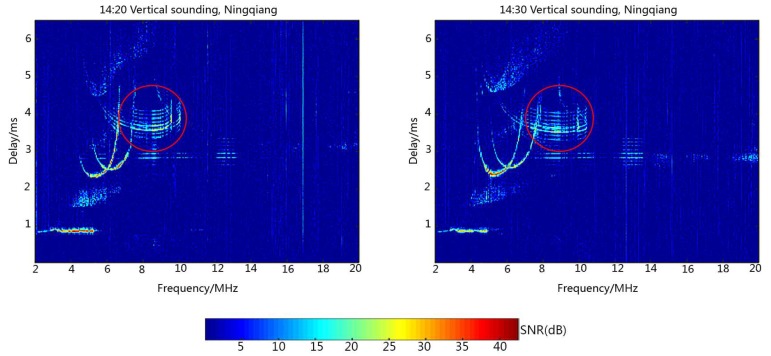
Multi-station sounding experiment using non-orthogonal complementary code.

In the second experiment, the ionosondes installed at Ningqiang and Wuhan synchronously transmitted the different codes, non-orthogonal complementary codes, at 16:00 BJT. Figure 6 shows the ionograms recorded at Ningqiang on 14 July 2018. Similar to Figure 5, Figure 6 also displays the vertical sounding echoes from Ningqiang and oblique sounding echoes (in red circles) from Wuhan. The signals received at Ningqiang are processed by the local-field emission code. Due to the non-orthogonal complementary codes, the receiver at Ningqiang also recorded the oblique signal transmitted from Wuhan. Similar to the first experiment, the ionograms in this case are also difficult to scale automatically using the current tool. This therefore inspires us to further change the transmitted codes in the experiment to avoid the interference resulting from the oblique sounding signal from Wuhan.

**Figure 7:**
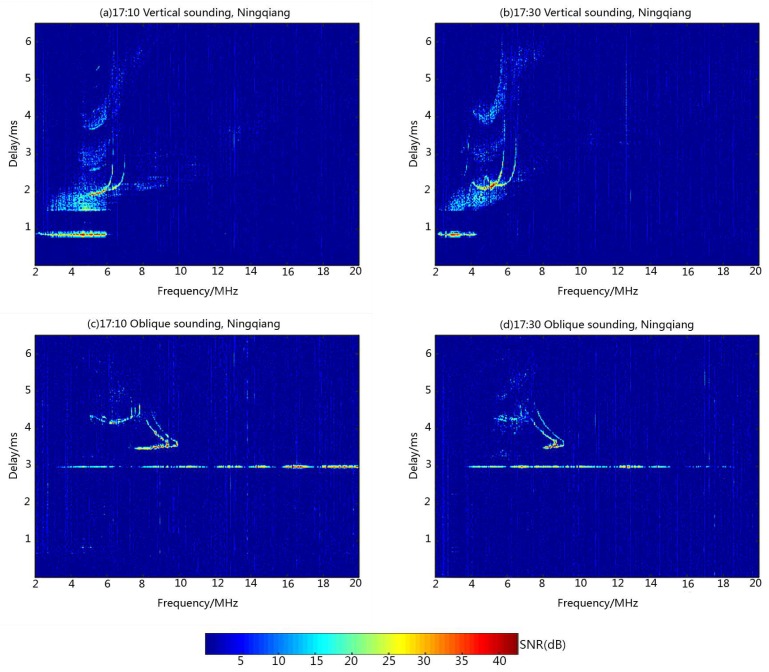
Multi-station sounding experiment using complete complementary code: Vertical sounding ionogram at (**a**) 17:10, and (**b**) 17:30; Oblique sounding ionogram at (**c**) 17:10, and (**d**) 17:30.

In the third experiment, the complete complementary code was applied for ionospheric sounding. The transmitted code was a {4, 4, 16} CCC, as shown in Table 1. The ionosondes at Ningqiang and Wuhan, respectively, transmitted the subsets A and B to carry out ionospheric sounding. Figure 7 shows the ionograms recorded at Ningqiang on 23 June 2018. Figure 7a,b correspond to the echoes processed by the subset A (vertical ionograms). Figure 7c,d correspond to the echoes processed by the subset B (oblique ionograms). Figure 7 clearly shows that the vertical and oblique echoes can be separated by use of different complete complementary code subsets. Based on the vertical ionograms, we can derive the virtual height of E and F2 layers in Ningqiang (h’E = 130 km, h’F2 = 290 km). Taking into account that the distance between Ningqiang and Wuhan is about 820 km, we can derive the ionospheric parameters in the midpoint of the propagation path according to the delay time in the oblique ionograms (h’E = 135 km, h’F2 = 310 km). Since the vertical and oblique echoes are distinguished by the matched filters of different subsets of CCC, the ionograms can be scaled automatically with the software.

## 6. Conclusions

In this study, a biphase CCC has been proposed for application to multi-station ionospheric sounding system to overcome the coherent interference. We have developed an algorithm of CCC generation and constructed the corresponding emission waveform for realistic experiments. Our experimental results at the HF band show that the coherent interference can be effectively eliminated in multi-station ionospheric sounding by using CCC. Moreover, the separation of vertical and oblique echoes on the single ionogram make it feasible for automatically scaling of ionograms in multi-station ionospheric sounding system. Aiming to eliminate the coherent interference in the network of radio systems, this research has the important implication to locate ionospheric irregularities, monitor travelling ionospheric disturbances (TIDs), and even enhance the global navigation satellite systems (GNSS).

However, the number of subsets and the number of subset sequences are the primary limiting factors for the application of the complete complementary code. Since the number of subsets cannot exceed the number of meta-code sequences, increasing the number of subset sequences therefore means the exponential growth of a single probe cycle, which may subsequently enlarge the detection dead zone and degrade the applicability of the complete complementary code.

## Figures and Tables

**Table 1:** {4, 4, 16} Complete Complementary Code.

A0	++++	+−+−	++−−	+−−+
A1	+−+−	++++	+−−+	++−−
A2	++−−	+−−+	++++	+−+−
A3	+−−+	++−−	+−+−	++++
B0	++++	−+−+	++−−	−++−
B1	+−+−	−−−−	+−−+	−−++
B2	++−−	−++−	++++	−+−+
B3	+−−+	−−++	+−+−	−−−−
C0	++++	+−+−	−−++	−++−
C1	+−+−	++++	−++−	−−++
C2	++−−	+−−+	−−−−	−+−+
C3	+−−+	++−−	−+−+	−−−−
D0	++++	−+−+	−−++	−++−
D1	+−+−	−−−−	−++−	++−−
D2	++−−	−++−	−−−−	+−+−
D3	+−−+	−−++	−+−+	++++

**Table 2:** Sidelobe peak level of ambiguity function with Doppler shift.

Waveform	0 Hz (dB)	5 Hz (dB)	10 Hz (dB)	20 Hz (dB)	100 Hz (dB)	200 Hz (dB)
31-bit m sequence	−6.59	−6.59	−6.59	−6.59	−6.63	−6.76
13-bit Barker code	−11.14	−11.14	−11.14	−11.14	−11.14	−11.11
16-bit complementary code	−	−28.24	−25.23	−22.15	−15.17	−12.13
{4, 4, 16} complete complementary code	−	−33.01	−30.00	−26.99	−19.96	−16.88

**Table 3:** Parameters of the emission waveform.

System Parameter	Typical Value	Rationale
Radiated power (W)	200	Required for adequate SNR
Receiver sensitivity (dBm)	−120	Keeps receiver noise below cosmic value
Pulse width (us)	25.6	Determines the range resolution
Pulse repetition interval (ms)	8.192	Provides the unambiguous range
Frequency range (MHz)	2~20	Required for the expected range of ionosphere
Frequency step (MHz)	0.05	Provides the electron destiny resolution of ionosphere
